# *ABCB1* variants and sex affect serotonin transporter occupancy in the brain

**DOI:** 10.1038/s41380-022-01733-1

**Published:** 2022-09-07

**Authors:** Leo R. Silberbauer, Lucas Rischka, Chrysoula Vraka, Annette M. Hartmann, Godber Mathis Godbersen, Cécile Philippe, Daniel Pacher, Lukas Nics, Manfred Klöbl, Jakob Unterholzner, Thomas Stimpfl, Wolfgang Wadsak, Andreas Hahn, Marcus Hacker, Dan Rujescu, Siegfried Kasper, Rupert Lanzenberger, Gregor Gryglewski

**Affiliations:** 1 Department of Psychiatry and Psychotherapy, Comprehensive Center for Clinical Neurosciences and Mental Health, Medical University of Vienna, Vienna, Austria; 2 Department of Biomedical Imaging and Image-guided Therapy, Division of Nuclear Medicine, Medical University of Vienna, Vienna, Austria; 3 Clinical Department of Laboratory Medicine, Medical University of Vienna, Vienna, Austria; 4 Center for Biomarker Research in Medicine (CBmed), Graz, Austria; 5 Center for Brain Research, Medical University of Vienna, Vienna, Austria; 6 Present Address: Child Study Center, Yale University, New Haven, CT USA

**Keywords:** Predictive markers, Neuroscience

## Abstract

Strategies to personalize psychopharmacological treatment promise to improve efficacy and tolerability. We measured serotonin transporter occupancy immediately after infusion of the widely prescribed P-glycoprotein substrate citalopram and assessed to what extent variants of the *ABCB1* gene affect drug target engagement in the brain in vivo. A total of 79 participants (39 female) including 31 patients with major depression and 48 healthy volunteers underwent two PET/MRI scans with the tracer [^11^C]DASB and placebo-controlled infusion of citalopram (8 mg) in a cross-over design. We tested the effect of six *ABCB1* single nucleotide polymorphisms and found lower SERT occupancy in *ABCB1* rs2235015 minor allele carriers (*n* = 26, MAF = 0.18) compared to major allele homozygotes (*t*_73_ = 2.73, *p*_FWE_ < 0.05) as well as in men compared to women (*t*_73_ = 3.33, *p*_FWE_ < 0.05). These effects were robust to correction for citalopram plasma concentration, age and diagnosis. From occupancy we derived the ratio of occupied to unoccupied SERT, because in theory this measure is equal to the product of drug affinity and concentration at target sites. A model combining genotype with basic clinical variables, predicted that, at the same dosage, occupied to unoccupied SERT ratio was −14.48 ± 5.38% lower in rs2235015 minor allele carriers, +19.10 ± 6.95% higher in women, −4.83 ± 2.70% lower per 10 kg bodyweight, and −2.68 ± 3.07% lower per 10 years of age. Our results support the exploration of clinical algorithms with adjustment of initial citalopram dosing and highlight the potential of imaging-genetics for precision pharmacotherapy in psychiatry.

## Introduction

Non-response to pharmacotherapy is a major challenge in the treatment of major depressive disorder (MDD). While roughly 30% percent of patients achieve remission, about 60% do not respond sufficiently to first-line treatment with selective serotonin reuptake inhibitors (SSRIs) [1, 2]. Moreover, a considerable number of patients experience adverse effects. Thus, most patients require modifications of their initial treatment [3]. Large-scale collaborative research projects pursue the characterization of predictors for treatment resistance that may guide initial treatment strategies [2]. Therapeutic drug monitoring can inform dose adaptations to achieve drug concentrations in blood associated with the highest possible probability of response and the lowest possible risk of adverse events. This approach may be supplemented by pharmacogenetic testing, such as determination of cytochrome P450 (CYP) genotype and consideration of basic pharmacokinetic variables [4]. While the cost-effectiveness of this approach has been demonstrated previously [5], predictive markers of individual response are highly anticipated to accelerate remission.

Binding of SSRIs to their molecular target, the serotonin transporter (SERT), correlates with their concentration in the brain and antidepressant efficacy may be associated therewith [6–8]. Next to plasma concentration, active efflux transport at the blood-brain barrier (BBB) might influence availability of antidepressants at target sites [9, 10]. While drug metabolism and basic pharmacokinetic variables directly affect plasma concentration, BBB permeability has been demonstrated to modify the association between plasma levels and cerebral concentration [11]. P-glycoprotein (P-gp), encoded by the ATP-binding cassette transporter B1 (*ABCB1*) gene, represents one of the principal efflux mechanisms at the BBB protecting the brain against potentially toxic xenobiotics [12, 13]. Among other pharmaceuticals, several psychotropic substances including SSRIs were shown to be substrates of the P-gp [10, 14]. (Es)citalopram is the most widely used SSRI [15, 16] and one of the strongest P-gp substrates within this drug class. Preclinical studies demonstrated 1.9–3.7 times higher citalopram brain concentrations in P-gp knockout mice after acute and chronic administration [17–21]. Therefore, genotyping of *ABCB1* holds promise as a predictive pharmacogenetic marker of antidepressant response based on the hypothesis that genetic variants might affect P-gp expression or function, and thus antidepressant concentration in the brain [9, 22]. Uhr et al. were the first to demonstrate an association between *ABCB1* genotype and clinical efficacy of antidepressants in MDD [18]. However, results of consecutive studies are equivocal and interpretation is hampered by methodological heterogeneity [23–35]. Inconsistencies may be explained by substrate specific effects of variants, disorder heterogeneity and non-uniform outcome definition. Besides clinical efficacy, a large body of evidence suggests that *ABCB1* genotype is associated with tolerability of antidepressants based on the assumption of genotype dependent alterations in cerebral drug accumulation (for review, see Brückl et al. [9]). Moreover, implications of *ABCB1* genotype in the treatment of other neuropsychiatric disorders, such as schizophrenia [36–38] and epilepsy [39] highlight its role whenever P-gp substrates are administered.

Nevertheless, progress in the determination of the utility of *ABCB1* genotyping by means of clinical trials is hampered by the need for large sample sizes and standardized treatment protocols. Adding to this, in light of the inherent heterogeneity of psychiatric disorders and the diversity of outcomes to define efficacy, the prospects of detecting and confirming relevant variants for each disorder and intervention appear distant. However, under the assumption that *ABCB1* variants affect cerebral drug availability, measurement of target engagement in the brain could be exploited as an intermediate phenotype to identify pharmacogenetic variants with trans-diagnostic relevance. Positron emission tomography (PET) enables the in vivo quantification of SERT occupancy, i.e., the proportion of transporter sites blocked by medication and, thus, may provide an indirect measure of cerebral drug availability. In this imaging-pharmacogenetics study, we aimed to assess the impact of *ABCB1* genotype on SERT occupancy by citalopram as a proxy of intracerebral drug availability in a large sample of healthy controls and patients diagnosed with MDD using gold standard SERT quantification procedures. Acute pharmacological challenge with intravenous citalopram was used to allow for the most direct assessment of the impact of efflux transport at the BBB on cerebral drug availability, because significant drug metabolism, first-pass effects, adaptations, and ceiling effects after prolonged therapy could be ruled out. This enabled the identification of variables with the potential to aid antidepressant dose adjustment and in this way contribute to precision pharmacotherapy in psychiatry.

## Methods

### Participants and study design

**Fig. 1:**
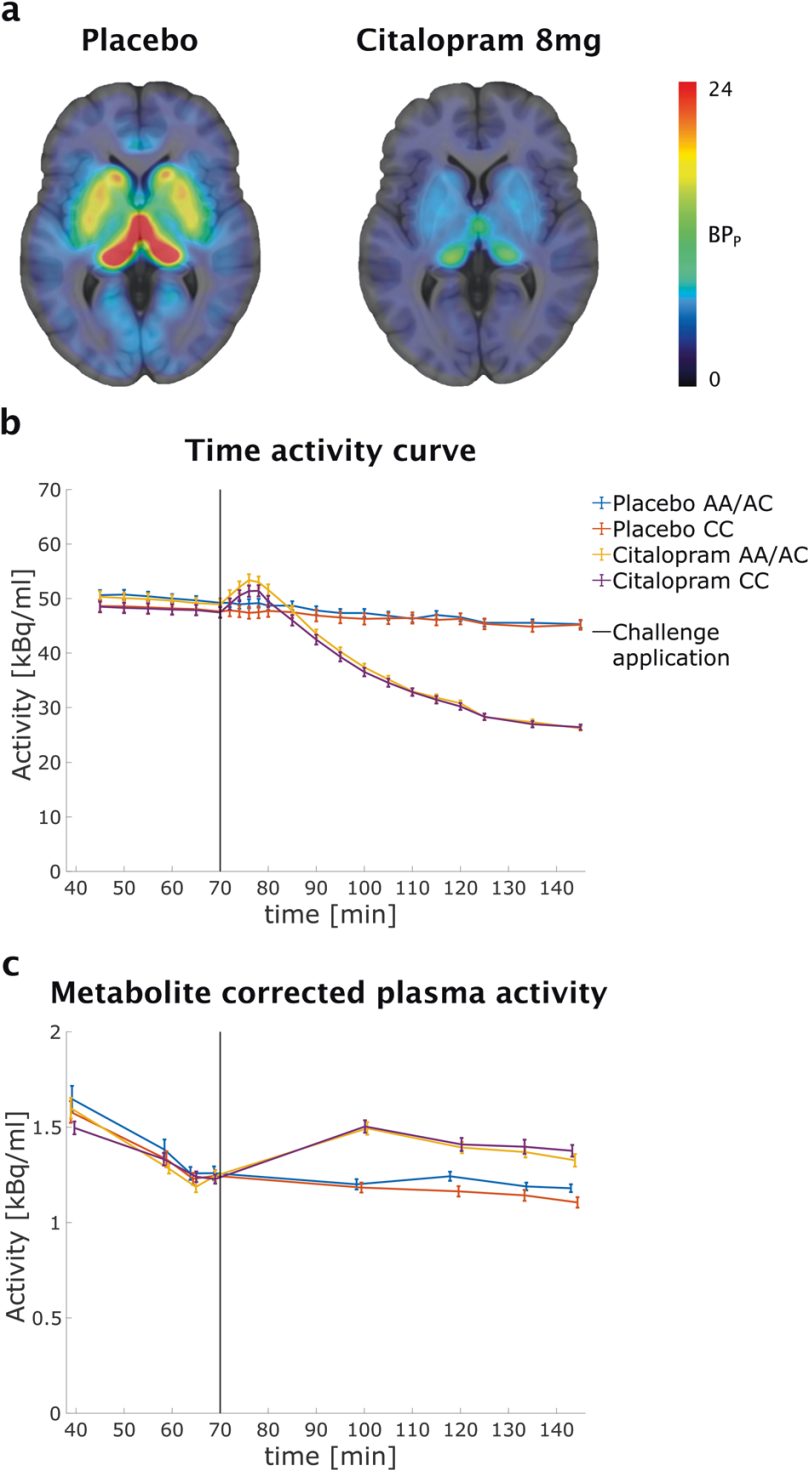
Quantification of serotonin transporter (SERT) binding potential (BP_P_). **a** Average SERT BP_P_ from 79 participants included in this study are displayed in transversal planes overlaid on a MR template for placebo (left) and citalopram (right) scans. High baseline binding was observed in regions rich in SERT such as thalamus and striatum. Time activity curves (**b**) for the thalamus and metabolite corrected plasma activity (**c**) ±SE for PET/MR scans with citalopram and placebo challenge are plotted for groups defined by *ABCB1*^rs2235015^ genotype.

Thirty-one patients with MDD (age ± SD: 29.0 ± 9.0) and 48 healthy volunteers (age ± SD: 28.0 ± 8.7, see Table 1) were included in these analyses. Participants underwent two PET/MR scans with the radioligand [^11^C]-N,N-dimethyl-2-(2-amino-4-cyanophenylthio)-benzylamine ([^11^C]DASB) during which double-blind intravenous pharmacological challenge with the SSRI citalopram (8 mg) or placebo was performed in a randomized cross-over study design (see Figure S1). Scanning procedures were performed using established protocols as detailed in the supplement [40]. In short, [^11^C]DASB was applied as bolus plus constant infusion according to a protocol designed to rapidly attain equilibrium in high-binding regions [41]. Good agreement with conventional bolus application protocols was demonstrated using this approach [42]. Drug or placebo infusion was performed over 8 min starting 70 min after initiation of radioligand application. Occurrence and severity of frequently observed side-effects to SSRIs were assessed during scans. Arterial blood samples were drawn throughout the measurement and assessment of the concentration of [^11^C]DASB, radioactive metabolites and citalopram in plasma was performed [41, 43]. Time activity curves and metabolite corrected plasma activity are displayed in Fig. 1.

**Table 1:** Demographics, clinical characteristics, pharmacokinetic and imaging parameters of study participants are shown.

	Diagnosis			*ABCB1*^rs2235015^		
	Controls	MDD	*p*	A + AC	*C*	*p*
Group size	48	31		26	53	
Age (y)	28.0 ± 8.7	29.0 ± 9.0	0.61^a^	29.0 ± 9.7	28.1 ± 8.3	0.69^a^
Sex (f/m)	24/24	15/16	1^b^	11/15	28/25	0.52^b^
Bodyweight (kg)	70.3 ± 12.0	68.8 ± 13.2	0.63^a^	70.1 ± 11.9	69.5 ± 12.8	0.83^a^
Citalopram AUC ((mg/ml) x sec)	94.4 ± 19.6	85.2 ± 21.0	0.06^a^	89.4 ± 20.1	91.5 ± 20.9	0.66^a^
HRSD	–	22.7 ± 5.1		–	–	
BDI	–	28.9 ± 8.2		–	–	
CGI	–	4.7 ± 0.7		–	–	
Placebo SERT BP_P_ (thalamus)	23.3 ± 4.2	24.2 ± 4.1	0.39^a^	23.5 ± 4.7	23.8 ± 3.9	0.78^a^
SERT occupancy (%)	67.3 ± 7.1	66.8 ± 4.9	0.75^a^	64.5 ± 6.4	68.4 ± 5.9	0.01^a^
O/U SERT	2.1 ± 0.7	2.1 ± 0.5	0.41^a^	1.9 ± 0.5	2.3 ± 0.6	<0.01^a^

Participants were grouped according to diagnosis (Controls/MDD) and *ABCB1*^rs2235015^ genotype (A + AC/C). Groups did not differ in terms of age, sex, bodyweight, AUC and imaging parameters. Minor allele carriers and major allele homozygotes did not differ in age, sex, bodyweight, AUC and baseline SERT binding potential. Significantly lower SERT occupancy was revealed in minor allele carriers when compared to major allele homozygotes.

*MDD* major depressive disorder, *A* *+* *AC* minor allele carriers, *C* major allele homozygotes, *HRSD* Hamilton Rating Scale for Depression, *BDI* Beck Depression Inventory, *CGI* Clinical Global Impression, *SERT* serotonin transporter, *BP*_*P*_ binding potential.

^a^Independent two-sample *t*-test.

^b^Chi-square test.

After completion of scanning procedures, antidepressant treatment with the SSRI escitalopram 10 mg (Cipralex, Lundbeck) was initiated in patients. Details on recruitment, inclusion criteria, clinical management and follow-up are outlined in the supplement.

### Genotyping and selection of single nucleotide polymorphisms (SNPs)

Eight *ABCB1* SNPs were considered for genotyping based on previously reported association with antidepressant treatment response to P-gp substrates or on association studies with *ABCB1* expression [18, 24, 27, 28, 30, 44–50]. Genotyping procedures are described in detail in the supplement [51, 52]. Genotype frequencies were found to be distributed according to the Hardy–Weinberg equilibrium (see Table 2). The following six tag SNPs (mean *r*^*2*^ = 0.97) were selected and used for further statistical analyses: rs1128503, rs2235015, rs10245483, rs28373093, rs2032583, rs1045642 (see Fig. S2). Participants were grouped into minor allele carriers and major allele homozygotes for all statistical analyses.

**Table 2:** Position of SNPs, minor allele frequency (MAF) and *p* value of Hardy–Weinberg statistics.

Polymorphism	Position	Minor allele (Major Allele)	MAF	Hardy–Weinberg *p* value
rs1045642	87509329	G(a)	0.48	0.52
rs2032583	87531245	G(a)	0.14	0.92
rs2032582	87531302	A(c)	0.39	0.81
rs2235033	87549827	A(g)	0.40	0.58
rs1128503	87550285	A(g)	0.38	0.99
rs2235015	87570248	A(c)	0.18	0.90
rs28373093	87714707	C(g)	0.42	0.46
rs10245483	90008294	G(t)	0.49	0.78

### Effect of *ABCB1* variants on SERT occupancy and clinical response

The thalamus was chosen for quantification of occupancy because our tracer application protocol is optimized for rapid equilibration in this region with high SERT expression [41]. Binding potentials (BP_P_) were obtained by subtracting the distribution volume of the reference region (cerebellar gray matter) from the thalamic distribution volume. SERT occupancy (ΔBP_P_) was calculated as the relative decrease in binding potentials between drug and placebo scans (see Fig. 1). R (v4.0.2, https://www.R-project.org/) was used for statistical analyses. To assess if *ABCB1* genotype affects SERT occupancy, multiple linear regression models were built for each tag SNP with participant’s age, sex, citalopram under the curve (AUC) in plasma and diagnosis as covariates. Age, sex and AUC were included based on pharmacokinetic assumptions, prior evidence of higher plasma levels in females and elderly patients and the association between SERT occupancy and SSRI plasma levels [11, 53–55]. Correction for family wise error (FWE) was performed using the Bonferroni method for six SNPs at alpha = 0.05. Furthermore, we performed mediation analyses using the PROCESS procedure to assess if effects of variables on SERT occupancy were mediated by citalopram plasma AUC. In an exploratory analysis, the effect of significant *ABCB1* variants and sex on treatment response (relative decrease in HDRS scores after six weeks of escitalopram treatment) was probed using independent *t*-tests. Pearson’s correlation coefficients were used to assess the association between SERT occupancy and treatment response. Moreover, we assessed the effect of genotype, sex and SERT occupancy on side-effect scores obtained during scans.

### Modeling SERT binding based on clinical variables and *ABCB1* genotype

While SERT occupancy (ΔBP_P_) facilitates comparison of our results with published findings, it asymptotically approaches 100% with increasing concentration, which hampers linear regression modeling and applicability of resulting models beyond the range covered by the data. Therefore, we calculated the ratio of occupied to unoccupied (O/U) SERT, which is directly derived from ΔBP_P_ for further analyses (Fig. S3), hereafter referred to as occupied SERT ratio or O/U SERT:

1 $${{{{{{{\mathrm{O}}}}}}}}/{{{{{{{\mathrm{U}}}}}}}}\;{{{{{{{\mathrm{SERT}}}}}}}} = \frac{\Delta{{{{{{{{\mathrm{BP}}}}}}}}_{{{{{{{\mathrm{P}}}}}}}}}}{{1-\Delta{{{{{{{\mathrm{BP}}}}}}}}_{{{{{{{\mathrm{P}}}}}}}}}} = \frac{\Delta{{{{{{{{\mathrm{BP}}}}}}}}_{{{{{{{{\mathrm{P}}}}}}}}({{{{{{{\mathrm{placebo}}}}}}}})}}}{\Delta{{{{{{{{\mathrm{BP}}}}}}}}_{{{{{{{{\mathrm{P}}}}}}}}({{{{{{{\mathrm{drug}}}}}}}})}}} - 1.$$

This alternative measure of drug target engagement can be used to approximate the product of drug affinity (1/*K*_i_) and free drug concentration at target sites ([I]) assuming ideal conditions as approached in vitro using equilibrium binding models and disregarding occupancy by endogenous ligands:

2 $${{{{{{{\mathrm{O}}}}}}}}/{{{{{{{\mathrm{U}}}}}}}}\;{{{{{{{\mathrm{SERT}}}}}}}}\sim \frac{{[{{{{{{{\mathrm{SERT}}}}}}}}.{{{{{{{\mathrm{I}}}}}}}}]}}{{[{{{{{{{\mathrm{SERT}}}}}}}}]}} = \frac{{\left[ {{{{{{{\mathrm{I}}}}}}}} \right]}}{{K_{{{{{{{\mathrm{i}}}}}}}}}}.$$

[SERT] and [SERT.I] signify the concentrations of free SERT and SERT-inhibitor complexes, respectively. The proportionality of O/U SERT to [I]/*K*_i_ enables modeling of linear effects of variables affecting SERT binding via changes in cerebral drug availability or affinity. A multiple linear regression model including weight, sex and age as predictors and occupied SERT ratio as the dependent variable was built. These basic parameters affecting pharmacokinetics are readily available and can be taken into account in clinical practice at the initiation of treatment. This model was compared to a model containing significant *ABCB1* variants using analysis of variance and the Akaike information criterion (AIC). For comparison, models calculated using SERT occupancy are included in the supplement.

## Results

### Effect of *ABCB1* variants on SERT occupancy and clinical response

**Fig. 2:**
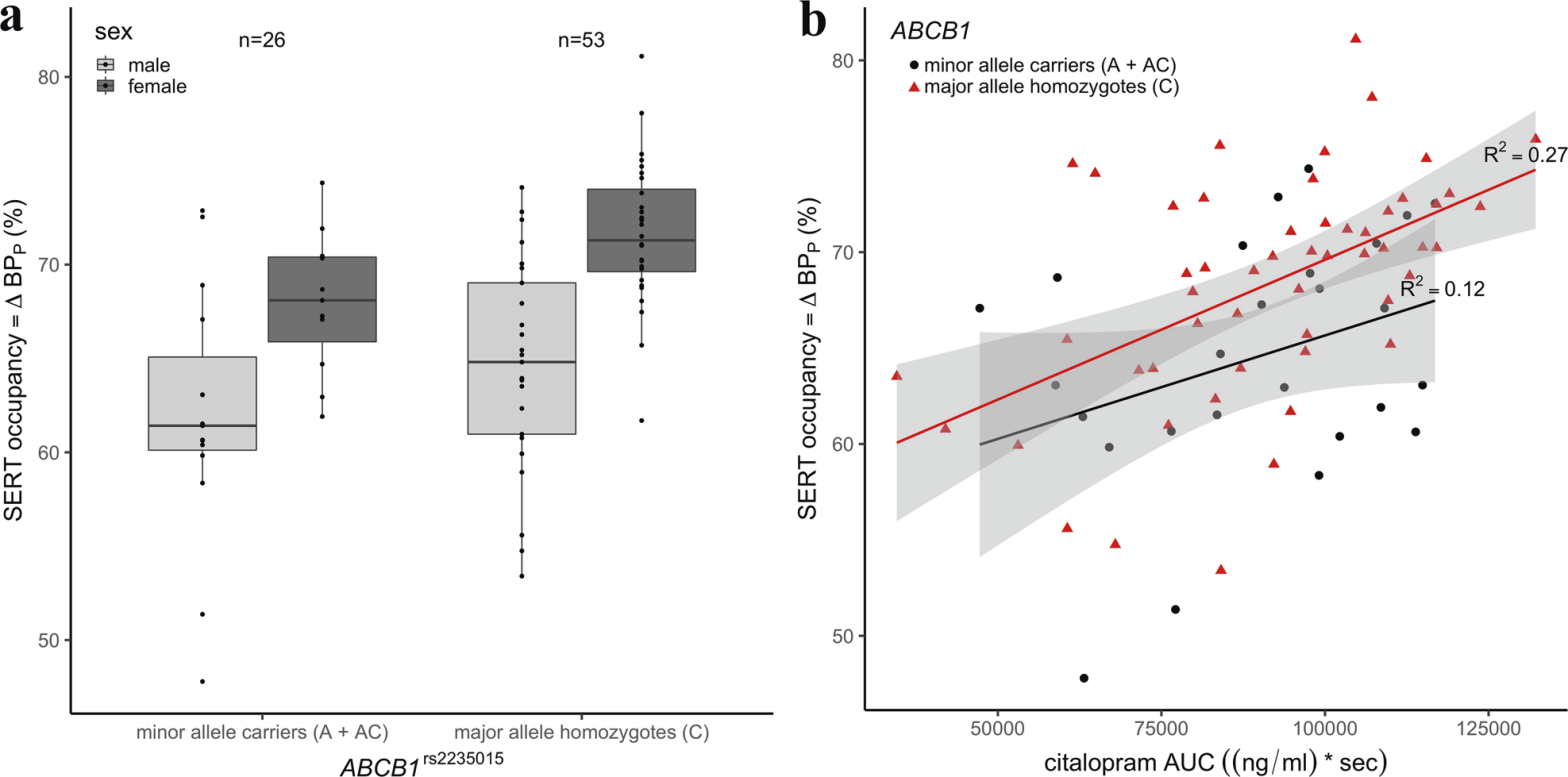
The effect of genotype, sex and citalopram plasma concentrtion on serotonin transporter occupancy. **a** Approximately one hour after infusion of 8 mg citalopram, lower serotonin transporter occupancy was observed in ABCB1^rs2235015^ minor allele carriers (A + AC) compared to major allele homozygotes (C) and female compared to male participants. **b** SERT occupancy is plotted against the area under the curve (AUC) of citalopram in plasma. Regression lines are plotted for participants grouped according to ABCB1^rs2235015^ genotype. Grey ribbons indicate 95% confidence intervals.

Demographics, clinical characteristics, pharmacokinetic and imaging parameters are listed in Table 1. Average SERT BP_P_ for placebo and citalopram condition are displayed in Fig. 1a. Regression analyses revealed a significant effect of rs2235015 on SERT occupancy (*t*_73_ = 2.73, *p*_FWE_ < 0.05) with lower occupancy in minor allele carriers compared to major allele homozygotes (Fig. 2a). This effect was robust to correction for citalopram plasma AUC (Fig. 2b), sex, age and diagnosis. Moreover, we detected a significant effect of sex with higher SERT occupancy in female compared to male participants (*t*_73_ = 3.33, *p*_FWE_ < 0.05). Notably, SERT BP_P_ was higher in female compared to male participants in our sample (mean ± SD: 25.56 ± 4.19 vs 21.80 ± 3.29). While the effect of rs2235015 was largely unaffected (*t*_72_ = 2.84, *p*_FWE_ < 0.05), the effect of sex on SERT occupancy was markedly decreased when additionally including placebo BP_P_ as a covariate (*t*_72_ = 2.03, *p* < 0.05, uncorrected). Results for non-significant SNPs are provided in the supplement (Table S1). Mediation analysis indicated a direct effect (*b* = −0.16, SE = 0.07) but no indirect effect of age mediated by citalopram AUC in plasma on SERT occupancy (indirect: −0.05, SE = 0.04). However, we found direct and indirect effects mediated by citalopram AUC in plasma of sex on SERT occupancy (direct: *b* = 5.47, SE = 1.33; indirect: *b* = 1.45, SE = 0.70). No significant effect of weight on SERT occupancy was detected after sex-wise mean centering.

In 25 patients with follow-up data, we observed a response rate of 68% (≥50% reduction in HDRS score) after six weeks of treatment with escitalopram. Antidepressant treatment response to escitalopram did not differ significantly between *ABCB1*^rs2235015^ minor allele carriers and major allele homozygotes. No association between sex and antidepressant response was revealed. Furthermore, we did not detect a significant correlation between SERT occupancy and antidepressant treatment response. Lastly, there was no statistically significant effect of *ABCB1*^rs2235015^ genotype, sex or SERT occupancy on side-effects measured during scans, which were not different between placebo and citalopram 8 mg (see supplement for further details).

### Modeling SERT binding based on clinical variables and *ABCB1* genotype

**Fig. 3:**
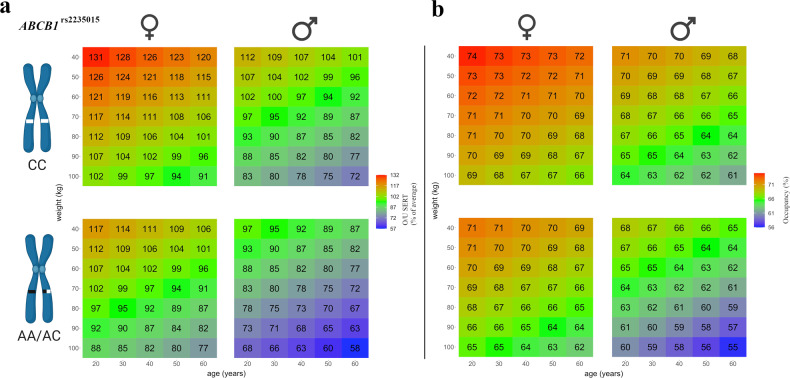
Modeling SERT binding based on clinical variables and *ABCB1* genotype. **a** The ratio of occupied to unoccupied (O/U) SERT after citalopram infusion was predicted for different combinations of sex, *ABCB1*^rs2235015^ genotype, age and weight based on a linear regression model fitted to the study data. O/U SERT is directly proportional to the product of drug affinity and concentration at target sites in equilibrium and is reported as a percentage of the sample’s average to aid interpretation of effects. O/U SERT was predicted to be −14.48 ± 5.38% lower in rs2235015 minor allele carriers, +19.10 ± 6.95% higher in women, −4.83 ± 2.70% lower per 10 kg bodyweight, and −2.68 ± 3.07% lower per 10 years of age. **b** Predictions of O/U SERT were transformed into SERT occupancy after citalopram 8 mg infusion based on equation 1 after multiplication with the sample’s mean O/U SERT.

In order to provide a model for estimating the effect of different clinical variables and *ABCB1* genotype on SERT binding, we transformed SERT occupancy into the ratio of occupied to unoccupied SERT (O/U SERT) which is proportional to the product of drug affinity and concentration at target sites. Effects are reported as percentages of the sample’s mean O/U SERT to improve interpretability. Adding rs2235015 to a model for prediction of O/U SERT using a combination of the basic clinical variables sex, age and weight improved the fit to the data (adjusted *R*^*2*^ = 0.35 vs. 0.30) with a lower AIC (722.98 vs 728.01). This model predicted that, at the same dosage, occupied SERT ratio was −14.48 ± 5.38% (SE) lower in rs2235015 minor allele carriers, +19.10 ± 6.95% higher in women, −4.83 ± 2.70% lower per 10 kg bodyweight, and −2.68 ± 3.07% lower per 10 years of age. Based on this model, predictions of occupied SERT ratio and SERT occupancy across different combinations of rs2235015 genotype, sex, weight and age are illustrated in Fig. 3. As an example, when comparing a 20-year-old female patient genotyped as rs2235015 major allele homozygote with a bodyweight of 50 kg with a 40-year-old rs2235015 male minor allele carrier with a bodyweight of 100 kg, O/U SERT in the female patient can be expected to be twice as high as in the male patient.

## Discussion

Research on the impact of *ABCB1* gene variants on in vivo brain concentration of psychopharmaceuticals is methodologically challenging and evidence is currently limited. Molecular neuroimaging studies investigating target occupancy may be used to indirectly assess the impact of patient characteristics on drug availability in the brain if drug affinity is not correlated with the variables of interest. Recently, Simoons et al. demonstrated significantly higher paroxetine SERT occupancy at the same plasma drug concentration after six weeks of therapy in *ABCB1* rs1128503 and rs2032582 minor allele carriers using [^123^I]β-CIT SPECT [11]. No association between SERT occupancy and rs1128503 genotype was found in our study. Increases in dopamine transporter binding following chronic paroxetine administration as shown in prior studies [56, 57] may hinder interpretation of results of the SPECT study considering non-selectivity of the applied radiotracer. Acute pharmacological challenge during PET measurements allows to neglect drug metabolism, first-pass and secondary effects, such as SERT up- and downregulation. Moreover, paroxetine applied in the SPECT study was demonstrated to be a relatively weak substrate of the P-gp while citalopram is one of the strongest P-gp substrates among SSRIs [58].

The intronic SNP rs2235015 is among the most frequently studied *ABCB1* SNPs in the context of antidepressant treatment [9]. Two studies support an association of rs2235015 with antidepressant response [18, 59]. Although these findings are in contrast to our results, the power for the investigation of the genetic impact on antidepressant response was limited due to the relatively high response rate and small sample with clinical follow-up. In the study by Uhr et al. rs2235015 minor allele carriers were more likely to remit after a six-week treatment trial with a P-gp substrate when compared to non-carriers [18]. Under the hypothesis of a direct relationship between target tissue drug concentration and treatment response, this finding is at odds with the results of our study that demonstrates lower SERT occupancy in minor allele carriers. This discrepancy may be interpreted in light of heterogeneous antidepressant treatment in the study by Uhr et al. and previously reported substrate-specific differences in the direction of effects of SNPs [30]. Moreover, direct comparisons of our results with those obtained after chronic oral drug administration neglects the influence of *ABCB1* variants on intestinal drug absorption [60]. *Breitenstein et al*. did not detect an association of rs2235015 genotype on treatment response but a significant genotype x plasma level interaction [59]. Interestingly, minor allele carriers had numerically higher antidepressant plasma concentrations when compared to non-carriers. An interpretation that might reconcile these findings with our results is that P-gp has a higher affinity for citalopram in rs2235015 minor allele carriers. This might result in higher efflux of citalopram at low concentrations, as observed after acute challenge of a low dose in our study, but lower efflux at high concentrations during chronic treatment due to a lower transport capacity of P-gp.

We detected a significant effect of sex on SERT occupancy. While previous studies did not assess sex specific differences in cerebral drug concentration, this finding is in line with known pharmacokinetic differences during antidepressant treatment between men and women [61–63]. Lower body weight and blood volume may account for higher plasma drug levels and, subsequently, higher cerebral concentration in women [63]. Mediation analysis indicated that a part of sex differences in SERT binding was due to differences in citalopram AUC in plasma. The effect of plasma concentration might be underestimated by using AUC, but equilibrium citalopram concentration in plasma was in many cases below the sensitivity of our quantification method. While gender-specific effects on CYP2C19 activity, the primarily responsible enzyme for degradation of citalopram, were previously reported, drug metabolism following intravenous administration can be neglected within our measurement timeframe. Sex differences in SERT occupancy were also associated with differences in placebo BP in our study. Although our results are in line with previously reported sex differences in SERT binding, more recent findings are equivocal [64, 65]. Higher BP in women in our study might have resulted in higher measures of occupancy due to regression to the mean [66]. The persistence of sex effects to correction for BP, indicates that there were sex differences in either cerebral citalopram concentration or SERT affinity. In line with our results, lower P-gp function was demonstrated in young women when compared to young men in a recent (R)-[^11^C]verapamil PET study [67].

Our findings may guide individual antidepressant dosing based on sex and *ABCB1* genotype. While several studies suggest a dose–response relationship, higher doses come with the cost of reduced tolerability [68]. Our results may reassure patients and clinicians that the practice of lower starting doses of citalopram and escitalopram to avoid initial side effects need not to come at the cost of SERT occupancy when performed in a targeted manner taking into account basic clinical variables and genotype. While the effects of dose adjustments need to be investigated in further clinical trials, this approach holds promise to reduce the rate of initial side effects and, thus, increase adherence during SSRI treatment. Our model employing occupied SERT ratio can be directly applied as a guidance in this process if assuming a linear relationship between drug dosage and concentration at target sites [69, 70]. As described above, O/U SERT ratio is expected to be directly proportional to the product of drug affinity and concentration. Furthermore, considering that citalopram is a competitive inhibitor of SERT, its effect on serotonin (5-HT) uptake in vitro is described by the following Michaelis-Menten equation (*K*_m_, Michaelis constant):

3 $$\frac{V}{{V_{m{{{{{{{\mathrm{ax}}}}}}}}}}} = \frac{{\left[ {5\mbox{-}{{{{{{{\mathrm{HT}}}}}}}}} \right]}}{{K_{{{{{{{\mathrm{m}}}}}}}}\left( {1 + \frac{{\left[ {{{{{{{\mathrm{I}}}}}}}} \right]}}{{K_{{{{{{{\mathrm{i}}}}}}}}}}} \right) + [5\mbox{-}{{{{{{{\mathrm{HT}}}}}}}}]}}.$$

This illustrates that the 5-HT concentration at which the half maximal velocity of SERT uptake (*V*) relative to the maximum rate (*V*_max_) is achieved is increased by a multiple of [I]/*K*_i_ in the presence of an inhibitor (I), which underlines the utility of O/U SERT as an outcome measure.

While our results may be applicable to drug dosing during the initiation of treatment, the effects of clinical variables and P-gp variants during prolonged treatment need to be established. For example, variants that lead to a higher affinity of P-gp and increased clearance of drug from the brain at low concentrations may lead to P-gp saturation at higher concentrations and thus inversely affect concentration in the brain during different phases of treatment [71].

Strengths of this investigation include the use of state-of-the-art PET imaging procedures that allow for highly specific and reliable SERT quantification in the context of a randomized, placebo-controlled trial. Participants were free from psychopharmacological medication, which allows for the investigation SERT occupancy in the absence of interactions. A relatively large sample of 79 participants was enrolled in this study which is seldom attained in resource intensive PET studies. Assessment of SERT occupancy following intravenous citalopram challenge allows for probing the genetic influence on active efflux transport at the BBB unaffected by drug metabolism and receptor up- and downregulation as observed during chronic treatment and, thus, is mainly dependent on cerebral drug availability and affinity [57].

Clinical implications of our findings remain to be determined in clinical trials with larger sample sizes. The assessed SNPs in our study were common (MAF ≥ 0.05), however, investigation of minor allele homozygotes could not be performed as group sizes did not provide sufficient statistical power. Generalizability of our results along a broader age spectrum need to be ascertained as mainly young participants were investigated in this trial. We observed differences in the arterial input function between genotypes. Based on the available literature we cannot provide a conclusive explanation for this phenomenon. However, preclinical data suggests that [^11^C]DASB does not interact with the P-gp at the BBB and, thus, no interferences with quantification of citalopram SERT occupancy are expected [72]. Nevertheless, possible interactions of [^11^C]DASB with the P-gp may be considered a limitation as in vivo data is currently lacking.

In conclusion, we demonstrate significant differences in SERT occupancy associated with *ABCB1* genotype and sex. Our study supports the further exploration of pharmacogenetic testing for *ABCB1* genotype during SSRI treatment. These results suggest that initial dosing of SSRI may be modified a priori based on patient characteristics and genotype, thus underscoring the potential of precision pharmacotherapy in psychiatry. While genetic testing of the *ABCB1* gene may be restricted to specialized centers, dose-adjustments based on sex could be performed easily in clinical practice.

## Supplementary information

Supplemental Material
