# Supplementary material for: ABCB1 variants and sex affect serotonin transporter occupancy in the brain
Source: Mol Psychiatry. 2022 Sep 7;27(11):4502–9. doi: 10.1038/s41380-022-01733-1 (PMC7613909; doi:10.1038/s41380-022-01733-1)
Supplement: Supplementary file 1 — Supplemental Material [file 41380_2022_1733_MOESM1_ESM.docx]

**ABCB1 variants and sex affect serotonin transporter occupancy in the brain**

***Supplemental information***

***Contents***

**Supplemental Text.** Supplementary methods…………………………...………………………..……………….2

**Supplemental Text**. Supplementary results….………………………………………………..…………………..7

**Supplemental Table S1.** Results from linear regression analyses for all tag SNPs………..……………………..8

**Supplemental Figure S1.** Consort Flow Diagram………………………………………………………………...9

**Supplemental Figure S2.** Linkage disequilibrium plot of genotyped SNPs…..……………………...…………10

**Supplemental Figure S3.** Ratio of occupied to unoccupied SERT …………..…………………………………11

**Supplemental References**………………………………………………………………………………………..12

**Supplementary methods**

Participants and study design

Participants were recruited at the Department of Psychiatry and Psychotherapy as well as via postings on dedicated message boards at the Medical University of Vienna, local supermarkets and electronic media. All participants were free from internal and neurological disease assessed via a thorough medical history, physical examination, electrocardiogram and routine laboratory parameters. The Structured Clinical Interview for DSM-IV for Axis I disorders (SCID-I) was applied to diagnose MDD in patients and to exclude the presence of any previous or current psychiatric diagnosis in healthy controls. Inclusion criteria for patients comprised a 17-item Hamilton Rating Scale for Depression (HRSD) score of ≥ 18. Patients had to be free from psychopharmacological treatment for at least three months prior to inclusion. Individuals had no history of substance use disorder and urine drug tests were performed at screening. In female participants, urine pregnancy testing was performed at screening and before each scan. Participants with contraindications for PET/MR scans including implants, claustrophobia and previous radiation exposure were excluded from participation. Participants were randomized to receive citalopram during either the first or second scan and placebo during the respective other scan. Randomization was performed by an independent researcher of our group not involved in study conduction and analysis. G*Power 3.1.9.2 estimated the power for detecting medium effect sizes (f²=0.15) using regression models with five predictors in our sample was at least 76.4% after FWE correction, not taking into account potential correlations between predictors that would increase power. All participants provided written informed consent and received financial reimbursement for their participation. All procedures were reviewed and approved by the ethics committee of the Medical University of Vienna and carried out according to the Declaration of Helsinki. This study was registered before the start of recruitment at clinicaltrials.gov (NCT02711215).

Clinical management and follow-up

Follow-up visits were performed in intervals of two weeks. Dosage was adapted according to clinical response defined as a minimum reduction of 50% in the HRSD scale. In case of non-response at the third follow-up visit patients were switched to 2nd line medication. Treatment response at the third visit was used for statistical analyses, as all patients received antidepressant monotherapy with escitalopram until this point. Four patients were lost to follow-up after imaging. For one participant, HRSD scores six weeks after initiation of SSRI treatment were missing and linearly interpolated from the visit before and after. Two patients were switched to 2^nd^ line medication due to intolerable adverse effects (sexual dysfunction in both participants) prior to the third follow-up visit. Ultimately, complete psychometric data during SSRI monotherapy was available in 25 patients.

Genotyping and selection of single nucleotide polymorphisms (SNPs)

Association of intragenic *ABCB1* SNPs with treatment response to antidepressant pharmacotherapy was demonstrated for rs1045642 [1-4], rs2032583 [5, 6], rs2032582 [7-10], rs2235033 [5], rs1128503 [11], rs2235015 [5]. Moreover, association with antidepressant treatment response was recently demonstrated for rs10245483, a putative promotor located approximately 2.5 Mb upstream of the *ABCB1* locus [12]. Higher P-glycoprotein expression was shown for minor alleles of the dinucleotide pair rs28373093/rs28656907 [13]. Nine ml Ethylene-Diamine-Tetraacetic-Acid (EDTA) blood samples were drawn from each participant. DNA was isolated from whole blood using the QIAamp DNA Blood Maxi Kit (Qiagen, Hilden, Germany). Genotyping was performed using the iPLEX assay on the MassARRAY Matrix-Assisted Laser Desorption/ Ionization Time-of-Flight (MALDI-TOF) mass spectrometer as described by Oeth et al. [14]. A set of primers comprising two PCR primers and one extension primer for each SNP was created using the software AssayDesign 3.1 (Sequenom, San Diego, USA). Evaluation of the obtained spectra was performed using the TyperAnalyzer v3.4.0.18 (Sequenom, San Diego, USA). Haploview version 4.2 (<http://www.broad.mit.edu/mpg/haploview/>) was used to test whether frequencies were according to the Hardy-Weinberg equilibrium and to calculate linkage disequilibrium (LD) among SNPs [15]. For the tri-allelic SNP rs2032582 the two most common alleles were applied.

The following quality criteria for genotyping were applied and met: Individual call rate > 85%, SNP call rate > 98%, identity of genotyped CEPH (Centre d'Etude du Polymorphisme Humain) samples with the expected genotype from the HapMap database [16]. The tagger algorithm implemented in Haploview version 4.2 was applied to select a subset of tag SNPs from all genotyped SNPs [17]. Tag SNPs capture the observed genetic variation within regions of strong linkage disequilibrium characterized by high levels of redundancy from one SNP to the next [18]. Using the pairwise tagging mode at a r^2^ threshold of ≥ 0.8 and a minimal allele frequency of 5% the following six tag SNPs were selected (mean r^2^=0.97): rs1128503, rs2235015, rs10245483, rs28373093, rs2032583, rs1045642 (see Figure S1).

PET/MR scanning procedures

Before each measurement, an arterial cannula was inserted in the radial artery for arterial blood sampling. Moreover, a venous cannula was inserted in a cubital vein of the opposite arm for administration of tracer and study drug. Synthesis and quality control of [^11^C]DASB was performed at the Department of Biomedical Imaging and Image-guided Therapy, Division of Nuclear Medicine, Medical University of Vienna as published previously [19]. Mean specific activity at the end of synthesis was 114.55 ± 87.47 Gbq/µmol. Application of [^11^C]DASB was initiated outside of the scanner and participants were transferred into the scanner after 30 min. A dosage of 17.78 ± 2.38 Mbq/kg was applied via an automated syringe pump. 20 milliliters were applied as bolus over 60 seconds, the rest was applied at a constant infusion rate of 7.4 ml/h, resulting in a K_bol_ of 162 min.

PET data was acquired continuously in list-mode for 125 min on a PET/MR scanner (Biograph mMR, Siemens Healthineers, Erlangen, Germany). Moreover, a structural T_1_-weighted image was acquired (MPRAGE, TE/TR=4.21/2000 ms, 1 x 1 mm in-plane resolution, 1 mm slice thickness, 0.1 mm gap). Reconstruction of list-mode PET data was performed using an ordinary Poisson-ordered subset expectation maximization algorithm (OP-OSEM, 3 iterations, 21 subsets). Attenuation correction was carried out using a low-dose CT scan (Siemens Biograph TruePoint PET/CT) that was recorded on a separate occasion, registered to T_1_-weighted MR data and scaled bilinearly to attenuation coefficients at 511 keV [20]. For one participant no low-dose CT could be obtained. Alternatively, a pseudoCT approach was applied [21] as a most accurate substitute [22]. For motion correction all frames were aligned to the mean image obtained from the longest period without head movement in visual inspection. PET data was co-registered to the T_1_-weighted MR image. The MR was normalized to Montreal Neurological Institute (MNI) space using SPM 12 (Wellcome Trust Center for Neuroimaging, London, United Kingdom) and the transformation matrix was applied to the PET data. Time-activity curves (TACs) were extracted from the thalamus and cerebellar grey matter excluding vermis and venous sinus as defined in the Automated Anatomical Labeling atlas [23] and reported previously [24].

70 min after initiation of tracer application double-blind pharmacological challenge with 8mg citalopram or saline was performed as continuous infusion over 8 min. The dose was chosen to ensure tolerability of acute drug challenge during scanning and in order to prevent ceiling effects observed at therapeutic doses [25]. The double-blind study medication was prepared by the hospital pharmacy of the Vienna General Hospital and was provided in syringes containing either citalopram or placebo diluted in saline. The study physician was provided with a sealed envelope containing the disclosure of the agent in case of adverse events requiring unblinding. Premature unblinding was not necessary in any participant. Citalopram rapidly distributes throughout the brain after intravenous infusion, as shown in rats [26] and evidenced by the instantaneous change in [^11^C]DASB binding in humans [27]. While tracer equilibrium may lag behind the equilibrium of citalopram, it is rapidly attained thereafter and clearance of citalopram is negligibly low and occurs at a half-life of approximately 24 h [28]. Therefore, citalopram concentration in the brain is expected to be relatively stable during scanning.

SERT quantification

Activity of regions of interest at equilibrium measured between 55 and 95 min after drug challenge (125 to 165 min after initiation of [^11^C]DASB application) was divided by the average metabolite-corrected plasma activity measured in up to three arterial samples drawn in that timeframe to obtain distribution volumes (V_T_). Binding potentials (BP_P_) were obtained by subtraction of the cerebellar distribution volume (V_ND_) from the distribution volume of the thalamus (V_T_) [29]:

(equation 1) BP_P_ = V_T_ – V_ND_.

Subsequently, citalopram SERT occupancy (ΔBP_P_) was calculated for each participant as the relative decrease in BP_P_ between conditions:

(equation 2) ΔBP_P_ = (BP_P-placebo_ – BP_P-drug_)/BP_P-placebo._

As occupancy asymptotically approaches 100% with increasing drug concentration, we calculated the ratio of occupied to unoccupied (O/U) SERT (short: occupied SERT ratio):

(equation 3) $O/U SERT$ = ΔBP_P_ / (1 – ΔBP_P_) = BP_P-placebo_ / BP_P-drug_ – 1.

We reported O/U SERT as a percentage of the sample’s mean to improve interpretability of effects:

(equation 4) ${(O/U SERT)}_{ri}=100*\frac{{(O/U SERT)}_{i}}{N}\sum_{i=1}^{N} {(O/U SERT)}_{i}$*.*

Assessment of citalopram plasma levels

Arterial blood plasma obtained from arterial samples drawn at 2, 5, 8, 12, 18, 30, 50, 60 and 70 min after initiation of study drug application was stored at -80°C prior to analysis. Citalopram concentrations were assessed with MassTox^®^ TDM Serie A test- kits (Chromsystems, Gräfeling, Germany) and liquid chromatography–tandem mass spectrometry (LC-MS/MS) at the Clinical Department of Laboratory Medicine, Medical University of Vienna. Successful pharmacological challenge was verified by assessment of citalopram plasma levels (mean ± SD peak plasma level=106.34 ± 22.06 ng/ml). The area under the curve (AUC) was calculated by integration of citalopram plasma levels using the AUCall function in the R-package PKNCA [31]. Fifty min after drug challenge 30.38% of participants had at least one sample below the sensitivity of the LC-MS/MS (< 7.8 ng/ml). Thus, AUC was used as it was considered a more reliable measure than plasma concentrations at individual time points before equilibration.

Assessment of side-effects

Occurrence and severity of frequently observed side-effects to SSRIs [32] were reported by participants on four-point Likert scales (I.E., 1-4) using a keypad after double-blind pharmacological challenge during scans. The following 13 items were assessed: nausea, restlessness, dizziness, discomfort, well-being, mood, anxiety, tension, alertness, tremor, sweating, dry mouth, headache. The total scores were calculated for each condition with higher scores indicating well-being or absence of side effects. For statistical analyses of tolerability, placebo total scores were subtracted from citalopram total scores to rule out effects associated with scanning procedures. Associations between tolerability and significant *ABCB1* variants and sex were probed with t-tests. Spearman correlation analysis was performed to assess the effect of SERT occupancy on tolerability. The Bonferroni method was used for FWE correction at alpha=0.05.

**Supplementary results**

Modeling SERT binding based on clinical variables and *ABCB1* genotype (SERT occupancy)

Adding rs2235015 to a model for prediction of SERT occupancy using a combination of the basic clinical variables sex, age and weight improved the fit to the data (adjusted R^2^=0.38 vs. 0.33) with a lower AIC (484.57 vs 489.92). Based on this model, predictions of SERT occupancy across different combinations of rs2235015 genotype, sex and age are illustrated in Figure 3. This model predicted that, at the same dosage, SERT occupancy was -3.28 ± 1.19% (standard error) lower in rs2235015 minor allele carriers, +4.09 ± 1.54% higher in women, -1.27 ± 0.60% lower per 10 kg bodyweight, and -0.80 ± 0.68% lower per 10 years of age.

Tolerability

No statistically significant difference in total scores of side-effects (t_150.01_=0.66, p=0.51) was observed between placebo (M=44.52, SD=6.01) and citalopram scans (M=43.95, SD=6.64). Tolerability did not differ significantly between *ABCB1*^rs2235015^ minor allele carriers and major allele homozygotes (t_49.92_=1.00, p_FWE_=0.96). No significant association between sex and tolerability was revealed (t_64.54_=1.73, p_FWE_=0.26). Lastly, we did not detect a significant correlation between SERT occupancy and tolerability (r=-0.19, p=0.12).

|  | **Estimate** | **SD** | **t** | **p** | **p_FWE_** |
| --- | --- | --- | --- | --- | --- |
| 1. **rs10245483**, adjusted R^2^ = 0.38 | | | | | |
| (Intercept) | 60.11 | 3.70 | 16.23 | 0.00 | 0.00 |
| rs10245483 | -0.41 | 1.36 | -0.30 | 0.77 | 4.60 |
| Age | -0.08 | 0.07 | -1.09 | 0.28 | 1.68 |
| Sex | 4.99 | 1.45 | 3.44 | 0.00 | 0.01 |
| AUC | 0.00 | 0.00 | 2.18 | 0.03 | 0.19 |
| Group | 0.38 | 1.24 | 0.31 | 0.76 | 4.56 |
| 1. **rs1128503**, adjusted R^2^ = 0.32 | | | | | |
| (Intercept) | 60.35 | 3.74 | 16.13 | 0.00 | 0.00 |
| rs1128503 | -0.44 | 1.23 | -0.36 | 0.72 | 4.33 |
| Age | -0.08 | 0.07 | -1.19 | 0.24 | 1.43 |
| Sex | 4.82 | 1.45 | 3.34 | 0.00 | 0.01 |
| AUC | 0.00 | 0.00 | 2.19 | 0.03 | 0.19 |
| Group | 0.43 | 1.23 | 0.35 | 0.73 | 4.38 |
| 1. **rs1045642**, adjusted R^2^ = 0.33 | | | | | |
| (Intercept) | 60.98 | 3.76 | 16.20 | 0.00 | 0.00 |
| rs1045642 | -1.42 | 1.38 | -1.03 | 0.31 | 1.85 |
| Age | -0.08 | 0.07 | -1.13 | 0.26 | 1.57 |
| Sex | 4.70 | 1.43 | 3.28 | 0.00 | 0.01 |
| AUC | 0.00 | 0.00 | 2.26 | 0.03 | 0.16 |
| Group | 0.53 | 1.23 | 0.43 | 0.67 | 4.00 |
| 1. **rs28373093**, adjusted R^2^ = 0.32 | | | | | |
| (Intercept) | 60.71 | 3.80 | 15.96 | 0.00 | 0.00 |
| rs28373093 | -0.80 | 1.29 | -0.62 | 0.54 | 3.23 |
| Age | -0.08 | 0.07 | -1.18 | 0.24 | 1.44 |
| Sex | 4.76 | 1.44 | 3.30 | 0.00 | 0.01 |
| AUC | 0.00 | 0.00 | 2.11 | 0.04 | 0.23 |
| Group | 0.42 | 1.23 | 0.34 | 0.73 | 4.40 |
| 1. **rs2032583**, adjusted R^2^ = 0.35 | | | | | |
| (Intercept) | 60.32 | 3.62 | 16.67 | 0.00 | 0.00 |
| rs2032583 | -2.48 | 1.32 | -1.88 | 0.06 | 0.39 |
| Age | -0.08 | 0.07 | -1.18 | 0.24 | 1.45 |
| Sex | 4.81 | 1.39 | 3.45 | 0.00 | 0.01 |
| AUC | 0.00 | 0.00 | 2.33 | 0.02 | 0.14 |
| Group | 0.93 | 1.24 | 0.75 | 0.45 | 2.72 |
| 1. **rs2235015**, adjusted R^2^ = 0.38 | | | | | |
| (Intercept) | 57.57 | 3.65 | 15.77 | 0.00 | 0.00 |
| rs2235015 | 3.28 | 1.20 | 2.73 | 0.01 | 0.05 |
| Age | -0.08 | 0.07 | -1.21 | 0.23 | 1.38 |
| Sex | 4.55 | 1.36 | 3.33 | 0.00 | 0.01 |
| AUC | 0.00 | 0.00 | 2.39 | 0.02 | 0.12 |
| Group | 0.92 | 1.19 | 0.77 | 0.44 | 2.65 |

**Supplemental Table S1** Results of regression analyses for all tag SNPs.

**Supplemental Figure S1** Consort Flow Diagram


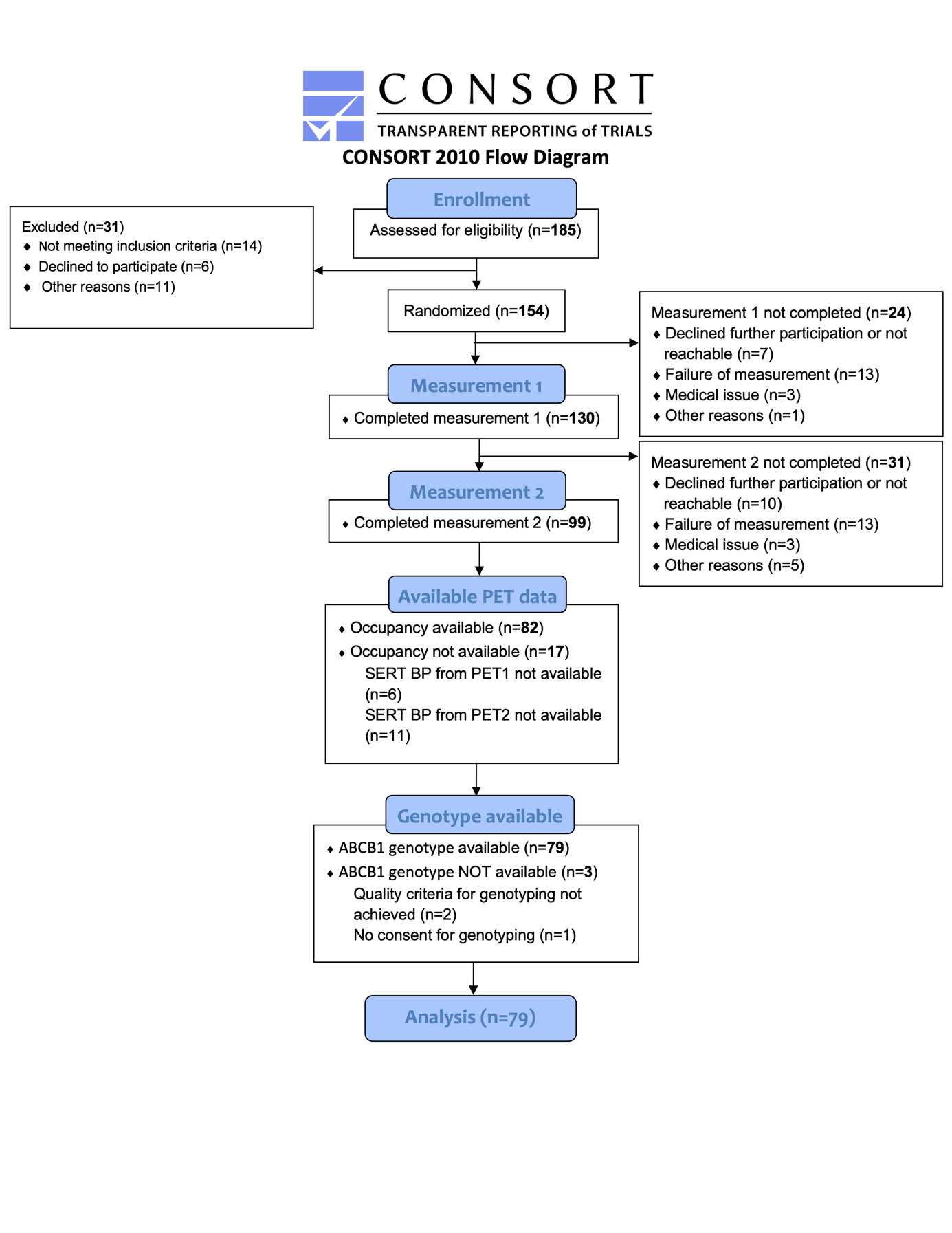


**Supplemental Figure S2** Linkage disequilibrium plot of genotyped single nucleotide polymorphisms (SNPs). Eight SNPs and the pairwise R^2^ between them are displayed. Relative positions of the SNPs to one another on the ABCB1 gene are depicted at the top. Below are the rs numbers for each SNP and the color scheme represents the strength of their R^2^ value. White = R^2^ = 0, shades of grey 0 < R^2^ < 1 and black = R^2^ = 1.


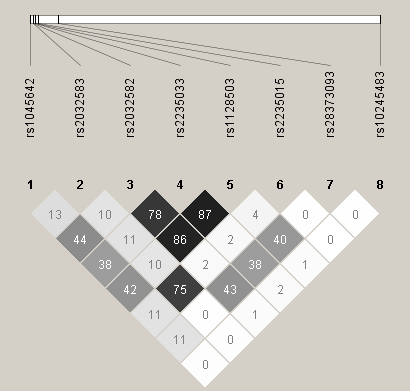


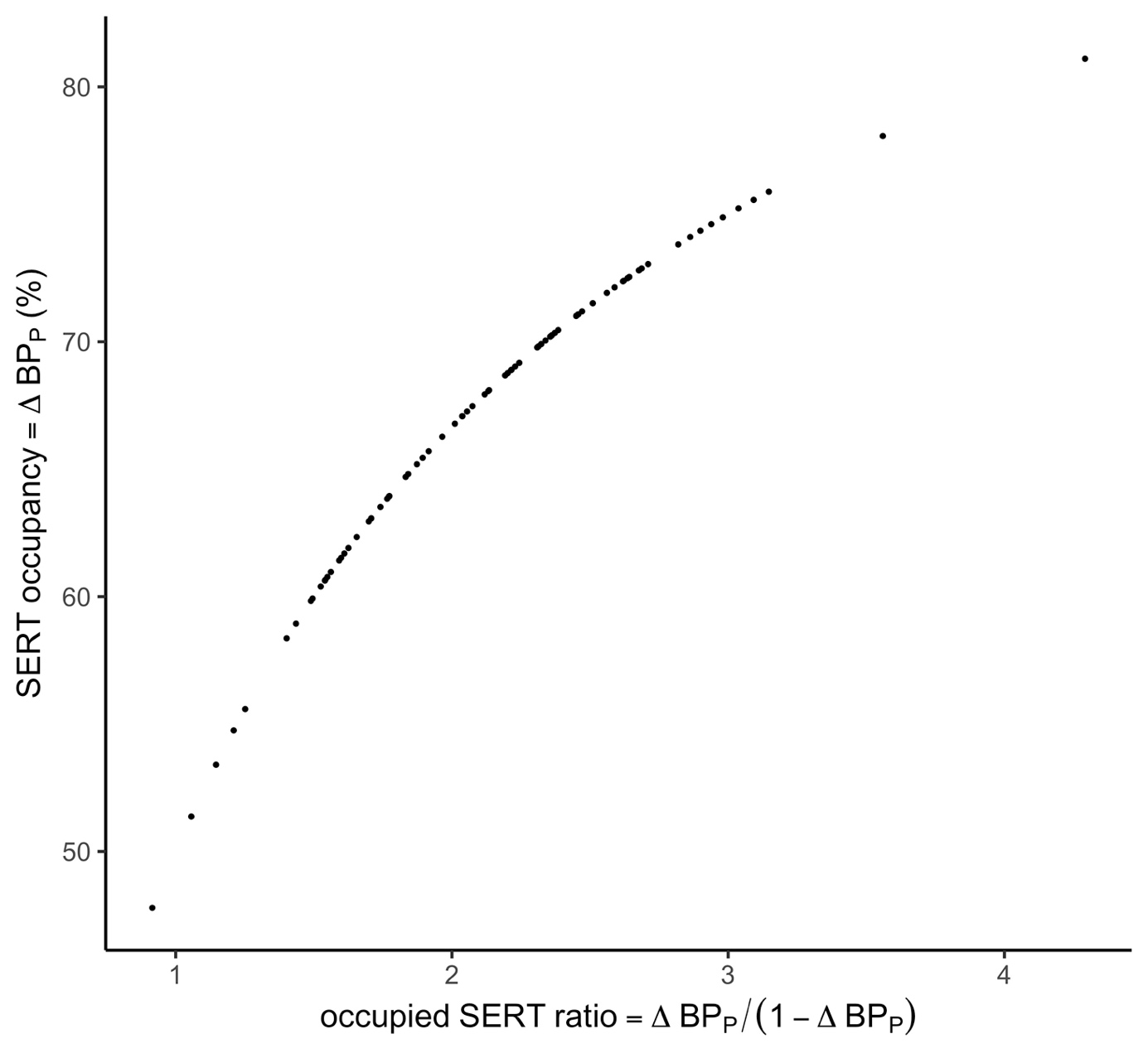


**Supplemental Figure S3** The ratio of occupied to unoccupied (O/U) SERT is calculated from SERT occupancy (ΔBP_P_). While ΔBP_P_ asymptotically approaches 100% with increasing drug concentration, O/U SERT is directly proportional to affinity and free drug concentration at target sites in equilibrium.

**Supplementary References**

1. Roberts RL, Joyce PR, Mulder RT, Begg EJ, Kennedy MA. A common P-glycoprotein polymorphism is associated with nortriptyline-induced postural hypotension in patients treated for major depression. *Pharmacogenomics J* 2002; **2**(3)**:** 191-196.

2. Lin KM, Chiu YF, Tsai IJ, Chen CH, Shen WW, Liu SC *et al.* ABCB1 gene polymorphisms are associated with the severity of major depressive disorder and its response to escitalopram treatment. *Pharmacogenet Genomics* 2011; **21**(4)**:** 163-170.

3. Singh AB, Bousman CA, Ng CH, Byron K, Berk M. ABCB1 polymorphism predicts escitalopram dose needed for remission in major depression. *Translational psychiatry* 2012; **2**(11)**:** e198.

4. Noordam R, Aarts N, Hofman A, van Schaik RH, Stricker BH, Visser LE. Association between genetic variation in the ABCB1 gene and switching, discontinuation, and dosage of antidepressant therapy: results from the Rotterdam Study. *J Clin Psychopharmacol* 2013; **33**(4)**:** 546-550.

5. Uhr M, Tontsch A, Namendorf C, Ripke S, Lucae S, Ising M *et al.* Polymorphisms in the drug transporter gene ABCB1 predict antidepressant treatment response in depression. *Neuron* 2008; **57**(2)**:** 203-209.

6. de Klerk OL, Nolte IM, Bet PM, Bosker FJ, Snieder H, den Boer JA *et al.* ABCB1 gene variants influence tolerance to selective serotonin reuptake inhibitors in a large sample of Dutch cases with major depressive disorder. *The Pharmacogenomics Journal* 2013; **13**(4)**:** 349-353.

7. Kato M, Fukuda T, Serretti A, Wakeno M, Okugawa G, Ikenaga Y *et al.* ABCB1 (MDR1) gene polymorphisms are associated with the clinical response to paroxetine in patients with major depressive disorder. *Progress in neuro-psychopharmacology & biological psychiatry* 2008; **32**(2)**:** 398-404.

8. Perroud N, Bondolfi G, Uher R, Gex-Fabry M, Aubry JM, Bertschy G *et al.* Clinical and genetic correlates of suicidal ideation during antidepressant treatment in a depressed outpatient sample. *Pharmacogenomics* 2011; **12**(3)**:** 365-377.

9. Gassó P, Rodríguez N, Mas S, Pagerols M, Blázquez A, Plana MT *et al.* Effect of CYP2D6, CYP2C9 and ABCB1 genotypes on fluoxetine plasma concentrations and clinical improvement in children and adolescent patients. *Pharmacogenomics J* 2014; **14**(5)**:** 457-462.

10. Chang HH, Chou CH, Yang YK, Lee IH, Chen PS. Association between ABCB1 Polymorphisms and Antidepressant Treatment Response in Taiwanese Major Depressive Patients. *Clinical psychopharmacology and neuroscience : the official scientific journal of the Korean College of Neuropsychopharmacology* 2015; **13**(3)**:** 250-255.

11. Bly MJ, Bishop JR, Thomas KL, Ellingrod VL. P-glycoprotein (PGP) polymorphisms and sexual dysfunction in female patients with depression and SSRI-associated sexual side effects. *J Sex Marital Ther* 2013; **39**(3)**:** 280-288.

12. Schatzberg AF, DeBattista C, Lazzeroni LC, Etkin A, Murphy GM, Jr., Williams LM. ABCB1 Genetic Effects on Antidepressant Outcomes: A Report From the iSPOT-D Trial. *Am J Psychiatry* 2015; **172**(8)**:** 751-759.

13. Loeuillet C, Weale M, Deutsch S, Rotger M, Soranzo N, Wyniger J *et al.* Promoter polymorphisms and allelic imbalance in ABCB1 expression. *Pharmacogenet Genomics* 2007; **17**(11)**:** 951-959.

14. Oeth P, Park C, Kosman D, Mistro G, van den Boom D, Jurinke C. iPLEX™ Assay: Increased Plexing Efficiency and Flexibility for MassARRAY System Through Single Base Primer Extension with Mass-Modified Terminators. 2005.

15. Barrett JC, Fry B, Maller J, Daly MJ. Haploview: analysis and visualization of LD and haplotype maps. *Bioinformatics* 2005; **21**(2)**:** 263-265.

16. Gibbs RA, Belmont JW, Hardenbol P, Willis TD, Yu F, Yang H *et al.* The International HapMap Project. *Nature* 2003; **426**(6968)**:** 789-796.

17. de Bakker PIW, Yelensky R, Pe'er I, Gabriel SB, Daly MJ, Altshuler D. Efficiency and power in genetic association studies. *Nat Genet* 2005; **37**(11)**:** 1217-1223.

18. Johnson GCL, Esposito L, Barratt BJ, Smith AN, Heward J, Di Genova G *et al.* Haplotype tagging for the identification of common disease genes. *Nat Genet* 2001; **29**(2)**:** 233-237.

19. Haeusler D, Mien LK, Nics L, Ungersboeck J, Philippe C, Lanzenberger RR *et al.* Simple and rapid preparation of [11C]DASB with high quality and reliability for routine applications. *Applied radiation and isotopes : including data, instrumentation and methods for use in agriculture, industry and medicine* 2009; **67**(9)**:** 1654-1660.

20. Carney JP, Townsend DW, Rappoport V, Bendriem B. Method for transforming CT images for attenuation correction in PET/CT imaging. *Medical physics* 2006; **33**(4)**:** 976-983.

21. Burgos N, Cardoso MJ, Thielemans K, Modat M, Pedemonte S, Dickson J *et al.* Attenuation correction synthesis for hybrid PET-MR scanners: application to brain studies. *IEEE transactions on medical imaging* 2014; **33**(12)**:** 2332-2341.

22. Rischka L, Gryglewski G, Berroterán-Infante N, Rausch I, James GM, Klöbl M *et al.* Attenuation Correction Approaches for Serotonin Transporter Quantification With PET/MRI. *Frontiers in Physiology* 2019; **10**(1422).

23. Tzourio-Mazoyer N, Landeau B, Papathanassiou D, Crivello F, Etard O, Delcroix N *et al.* Automated anatomical labeling of activations in SPM using a macroscopic anatomical parcellation of the MNI MRI single-subject brain. *NeuroImage* 2002; **15**(1)**:** 273-289.

24. Lanzenberger R, Kranz GS, Haeusler D, Akimova E, Savli M, Hahn A *et al.* Prediction of SSRI treatment response in major depression based on serotonin transporter interplay between median raphe nucleus and projection areas. *NeuroImage* 2012; **63**(2)**:** 874-881.

25. Meyer JH, Wilson AA, Sagrati S, Hussey D, Carella A, Potter WZ *et al.* Serotonin transporter occupancy of five selective serotonin reuptake inhibitors at different doses: an [11C]DASB positron emission tomography study. *Am J Psychiatry* 2004; **161**(5)**:** 826-835.

26. Bundgaard C, Jørgensen M, Mørk A. An integrated microdialysis rat model for multiple pharmacokinetic/pharmacodynamic investigations of serotonergic agents. *J Pharmacol Toxicol Methods* 2007; **55**(2)**:** 214-223.

27. Gryglewski G, Klobl M, Berroteran-Infante N, Rischka L, Balber T, Vanicek T *et al.* Modeling the acute pharmacological response to selective serotonin reuptake inhibitors in human brain using simultaneous PET/MR imaging. *Eur Neuropsychopharmacol* 2019.

28. Sogaard B, Mengel H, Rao N, Larsen F. The pharmacokinetics of escitalopram after oral and intravenous administration of single and multiple doses to healthy subjects. *Journal of clinical pharmacology* 2005; **45**(12)**:** 1400-1406.

29. Innis RB, Cunningham VJ, Delforge J, Fujita M, Gjedde A, Gunn RN *et al.* Consensus nomenclature for in vivo imaging of reversibly binding radioligands. *Journal of cerebral blood flow and metabolism : official journal of the International Society of Cerebral Blood Flow and Metabolism* 2007; **27**(9)**:** 1533-1539.

30. HILL M, LONDON N. Proceedings of the physiological society. *measurement* 1953; **16:** 17.

31. Denney W, Duvvuri S, Buckeridge C. Simple, Automatic Noncompartmental Analysis: The PKNCA R Package. *Journal of Pharmacokinetics and Pharmacodynamics* 2015; **42:** S65-S65.

32. Seifritz E, Baumann P, Müller MJ, Annen O, Amey M, Hemmeter U *et al.* Neuroendocrine effects of a 20-mg citalopram infusion in healthy males. A placebo-controlled evaluation of citalopram as 5-HT function probe. *Neuropsychopharmacology : official publication of the American College of Neuropsychopharmacology* 1996; **14**(4)**:** 253-263.
